# Total unilateral pulmonary collapse secondary to allergic bronchopulmonary aspergillosis: a case series of an unusual cause of complete atelectasis

**DOI:** 10.1186/s12890-021-01789-9

**Published:** 2021-12-24

**Authors:** N. Benkalfate, S. Dirou, P. Germaud, C. Defrance, A. Cavailles, T. Pigeanne, M. Robert, T. Madjer, F. Corne, L. Cellerin, C. Sagan, F. X. Blanc

**Affiliations:** 1 Nantes Université, CHU Nantes, Service de Pneumologie, L’Institut du thorax, Nantes, 44000 France; 2 Nantes Université, CHU Nantes, Service de Radiologie et d’Imagerie Médicale, Unité d’Imagerie Thoracique et Générale, Nantes, 44000 France; 3 Service de Pneumologie, Centre Hospitalier Côte de Lumière, Les Sables d’Olonne, 85340 France; 4 Consultation de Pneumologie, Clinique Jules Verne, Nantes, 44300 France; 5 Nantes Université, CHU Nantes, Service d’Anatomopathologie, Nantes, 44000 France

**Keywords:** Allergic bronchopulmonary aspergillosis, Lung collapse, Complete atelectasis

## Abstract

**Background:**

Allergic bronchopulmonary aspergillosis (ABPA) is a bronchopulmonary disease caused by a complex hypersensitivity to *Aspergillus* and is usually associated with underlying respiratory diseases such as asthma or cystic fibrosis. Mucus plugging can lead to segmental or lobar atelectasis, but complete lung atelectasis has been exceptionally reported in the literature, making it difficult to diagnose. The diagnosis of ABPA may however be suggested in patients without known predisposing respiratory disorder, even in the absence of other relevant radiographic findings.

**Case presentation:**

We report five cases of total unilateral lung collapse secondary to ABPA in 70–81-year-old women. Two of them had a past history of ABPA, while total unilateral lung collapse was the first sign of the disease in the other three patients, contributing to the initial misdiagnosis. Flexible bronchoscopy was initially performed to remove mucus plugs from the obstructed airways but was inefficient in four cases. Corticosteroid and/or antifungal treatment was needed.

**Conclusion:**

ABPA can cause total unilateral lung collapse even in patients without known underlying chronic respiratory disease, making the diagnosis difficult. Flexible bronchoscopy should be considered when lung collapse is associated with respiratory distress but corticosteroids are the mainstay treatment for ABPA.

## Background

Allergic bronchopulmonary aspergillosis (ABPA) is an immune-mediated inflammatory disease resulting from a complex hypersensitivity reaction to bronchial colonization by *Aspergillus* spp. [1]. It can be defined as an aberrant immune response against *Aspergillus* leading to a lymphocyte response and to immediate immunoglobulin E (IgE)-mediated hypersensitivity to *Aspergillus* [2]. It is the most common *Aspergillus*-related disorder and usually affects patients with a preexisting respiratory disorder such as asthma or cystic fibrosis [3–5]. The radiographic findings associated with ABPA usually include proximal or distal mucoid impaction, pulmonary infiltrates or condensations, and centrolobular nodules [6–8]. Mucus plugs can lead to segmental or lobar atelectasis, and are more often described in the literature [6–8]. However, complete lung atelectasis due to ABPA is extremely rare with only a few cases reported in the literature [9–16], so that specific therapeutic guidelines are lacking.

Here, we reported five cases of total unilateral lung collapse caused by ABPA in patients without known respiratory disorders. All the patients were hospitalized in Nantes University Hospital (‘CHU Nantes’), France. In this series, the International Society for Human and Animal Mycology (ISHAM) Working Group criteria [17] and the new clinical diagnostic criteria for ABPA/allergic bronchopulmonary mycosis (ABPM) proposed by Asano et al [18] were used to make the diagnosis of ABPA. The optimal management of these patients was discussed, based on our own experience and on the description of other cases reported since 1982, and we raised specialists’ awareness about this uncommon presentation of ABPA that may be life-threatening in case of respiratory distress.

A literature review was performed in January 2021. The terms “aspergillosis”, “ABPA”, “lung collapse” and “atelectasis” were searched in PubMed. Clinical reports of cases of total unilateral lung collapse secondary to ABPA were included.

## Case presentation

### Case 1

A 70-year-old woman without any past history of asthma or cystic fibrosis had been diagnosed with ABPA in 1999. In April 2008, she presented with dry and non-productive cough associated with increased dyspnea. The laboratory tests showed a normal eosinophil count (210/mm^3^), normal C-reactive protein (CRP) levels, total IgE levels increased from 200 to 791 kU/L, elevated *Aspergillus fumigatus*-specific IgE levels (8.05 kU/L) and positive results for *Aspergillus fumigatus* precipitins (4 precipitin bands). Chest imaging showed total collapse of the left lung.

Flexible bronchoscopy was performed twice but failed to completely remove all bronchial mucus plugs. The analysis of the collected mucus plugs allowed detecting *Aspergillus fumigatus* in both direct examination and culture. The patient was treated with corticosteroids for six weeks and itraconazole for six months. One month later, the clinical presentation was improved and the chest X-ray was normalized. Disease remission was maintained and she did not experience any recurrent exacerbation to date.

### Case 2

**Fig. 1:**
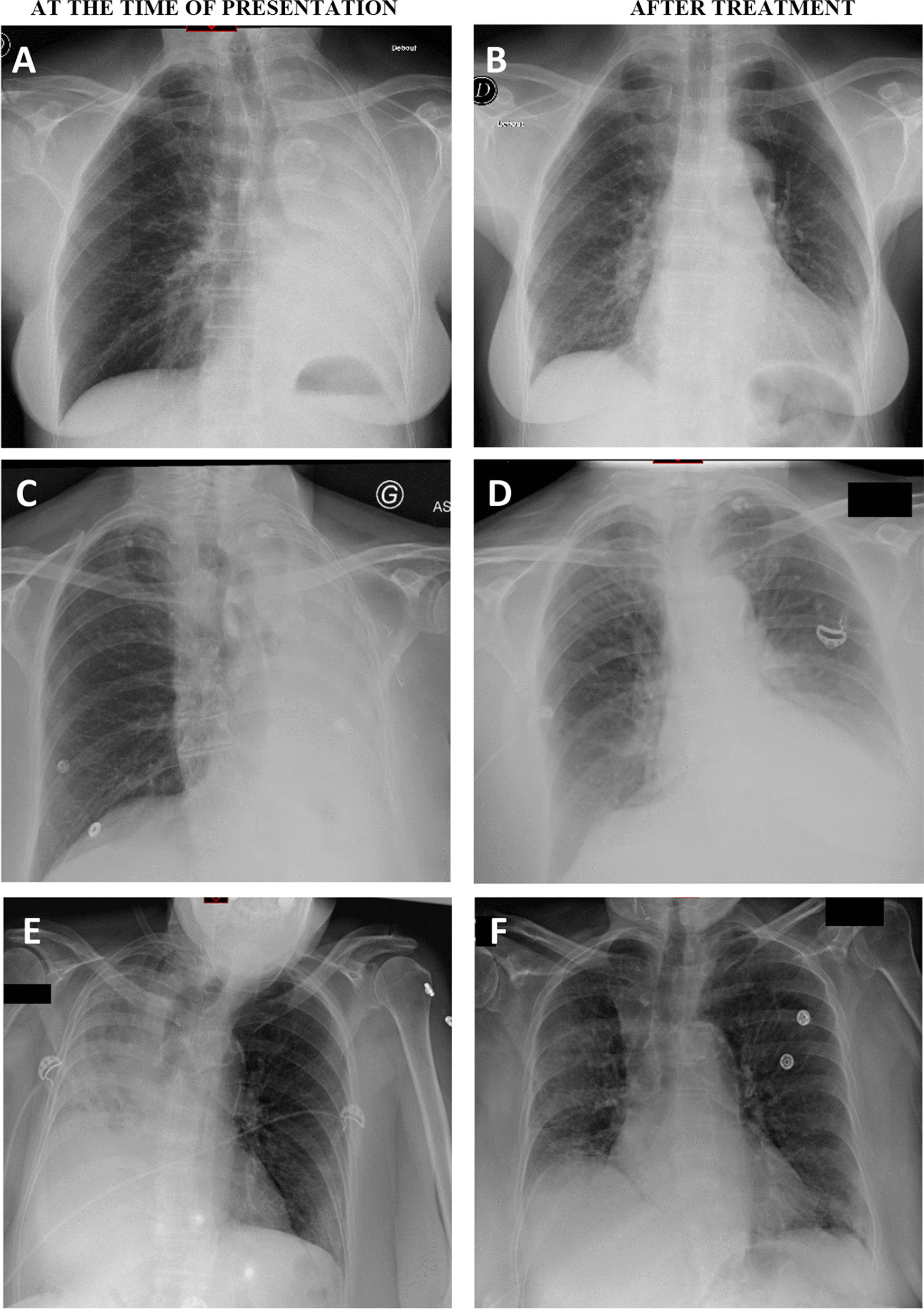
Chest X-ray of case #2 (**A**, **B**), #4 (**C**, **D**), and #5 (**E**, **F**) at the time of initial presentation, showing total lung atelectasis (**A**, **C**, **E**), and after treatment with flexible bronchoscopy and corticosteroids (**B**, **D**, **F**), showing a significant improvement

A 74-year-old woman without any significant history of pulmonary disease reported cough and rhinorrhea since last winter. In June 2013, she complained of productive cough and very thick sputum associated with left chest pain, anorexia and asthenia. No improvement was noted after two lines of antibiotics, so that a chest X-ray was performed and showed total collapse of the left lung (Fig. 1A). The thoracic computed tomography (CT) scan showed bronchiectasis, a mucus plug in the middle lobe and mucus plugs in the left central bronchus. Laboratory tests showed hypereosinophilia (2500/mm^3^), elevated total IgE levels (3364 kU/L) and positive results for *Aspergillus fumigatus*-specific IgG (94 kUA/L) and IgE (62.6 kU/L). The skin prick test to *Aspergillus fumigatus* antigen was positive.

The patient underwent flexible bronchoscopy that failed to remove the mucus plugs. Medical treatment with corticosteroids was initiated and itraconazole was also given for 9 months. After 10 days of corticosteroids, flexible bronchoscopy was repeated and numerous plugs were aspirated. The chest X-ray was normalized (Fig. 1B). *Aspergillus fumigatus* was found in the endoscopic samples. A home environment assessment showed a damp house with high concentrations of *Aspergillus fumigatus*. To date, the patient did not report any recurrent exacerbation.

### Case 3

A 73-year-old woman without underlying pulmonary disease complained of asthenia with chronic cough for several months and expectoration of dark brown mucus plugs. Due to the absence of improvement after two lines of antibiotics, a chest CT scan was performed and revealed total left lung collapse in the absence of other findings consistent with ABPA in October 2014.

**Fig. 2:**
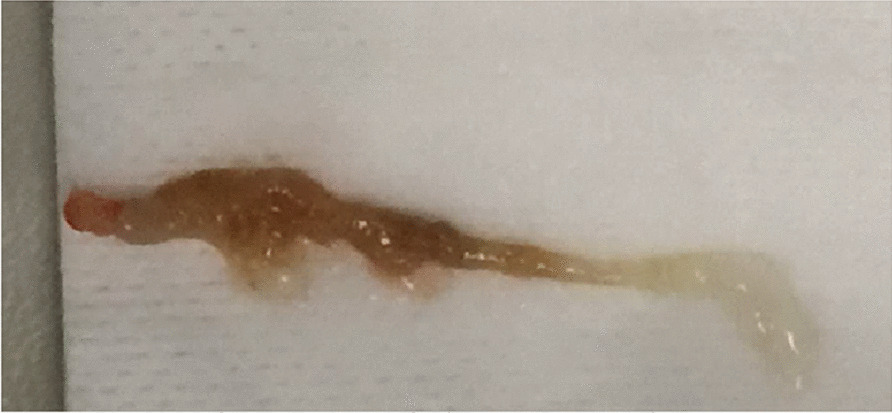
Bronchial mold removed during rigid bronchoscopy (case #3)

Initial flexible bronchoscopy revealed a tumor that could not be mobilized in the left bronchus, suggesting an underlying lung cancer. One week later, rigid bronchoscopy allowed removing a giant mucus plug and the atelectasis (Fig. 2). It contained numerous Charcot-Leyden crystals and septate hyphae. Total IgE and *Aspergillus fumigatus*-specific IgE levels (406 kU/L and 10.3 kUA/L, respectively) were elevated. The results for *Aspergillus fumigatus-*specific IgG were negative and the eosinophil count was moderately high (800/mm^3^). One month later, chest radiography was normalized. The patient was treated with itraconazole but treatment was discontinued 2 months later due to the occurrence of hepatic cytolysis. Oral corticosteroids were resumed for 6 weeks. The patient did not experience any recurrent exacerbation thereafter.

### Case 4

A 72-year-old woman without any underlying pulmonary disorder was diagnosed with ABPA in 1998. Her last exacerbation occurred in 2008. In December 2016, she experienced dry cough for 1 month with no improvement after two lines of antibiotics. A chest X-ray was performed and showed complete atelectasis of the left lung and tram-track opacities in the right lower field (Fig. 1C). Total IgE and *Aspergillus fumigatus*-specific IgE levels (2442 kU/L and 56.6 kUA/L, respectively) were elevated. The results for *Aspergillus fumigatus*-specific IgG were positive and the eosinophil count were negative while oral corticosteroids were initiated a few days earlier.

Flexible bronchoscopy showed a large mucus plug that was partially removed after suction. *Aspergillus fumigatus* was found in the endoscopic samples. Itraconazole and corticosteroids were initiated. A few days later, the symptoms worsened, resulting in respiratory distress for which intubation and mechanic ventilation were needed. Flexible bronchoscopy was repeated and the use of a cryoprobe to freeze the plug allowed its removal. Medical treatment was continued and the patient was rapidly extubated. Two months later, she reported a significant improvement in cough and the chest X-ray showed a full resolution (Fig. 1D).

### Case 5

**Fig. 3:**
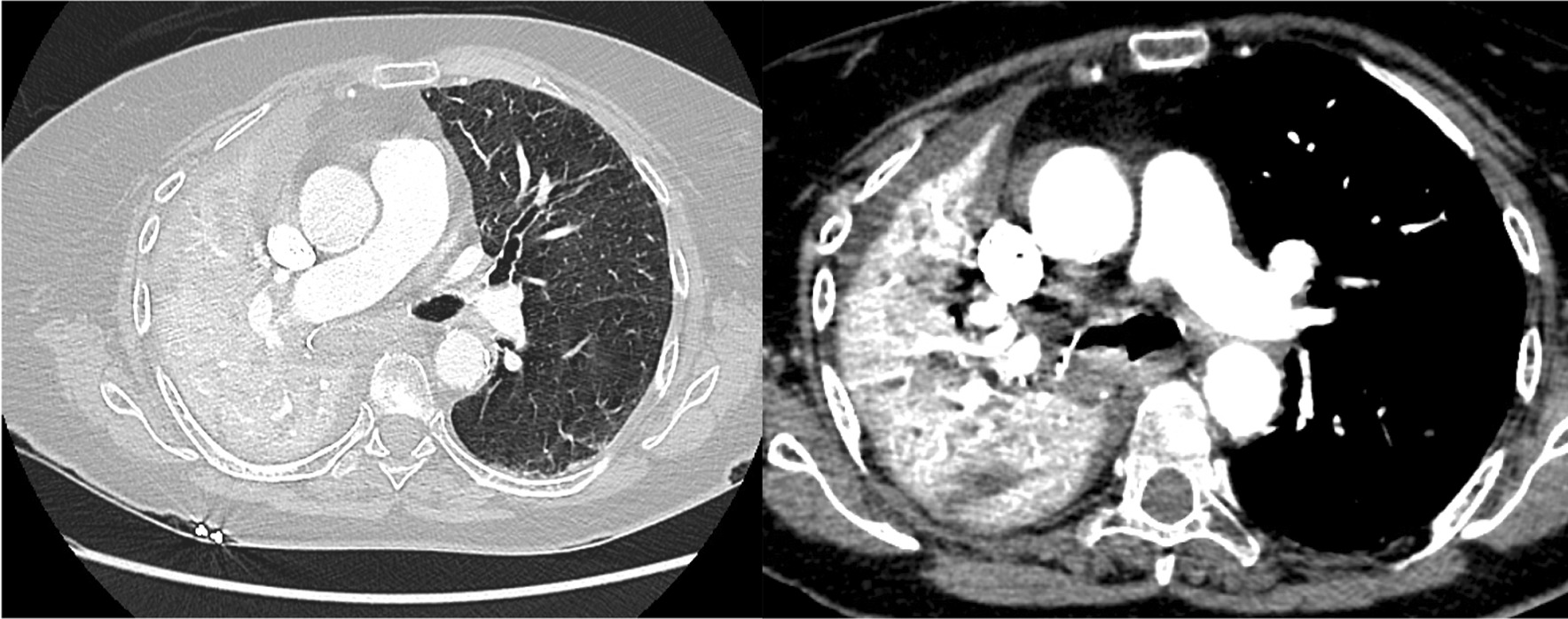
High-resolution computed tomography showing total right lung collapse (case #5)

An 81-year-old former smoker woman was followed since 2013 for chronic bronchitis with respiratory symptoms, including wheezing. She was hospitalized in December 2020 for productive cough and dyspnea for over a week. A chest X-ray showed total right lung collapse (Fig. 1E). Based on her smoking history, a lung cancer was first suspected. A CT scan was performed and showed pulmonary embolism and confirmed the presence of right lung atelectasis due to the right main bronchus obstruction without any other finding supporting an underlying neoplastic process (Fig. 3). Her condition deteriorated rapidly with respiratory distress for which emergency flexible bronchoscopy was needed. Bronchoscopy showed that the right main bronchus was obstructed by a thick and large mucus plug that could be completely removed. Her condition improved rapidly thereafter and chest radiography no longer showed atelectasis (Fig. 1F). Total IgE and *Aspergillus fumigatus*-specific IgE levels (2464 kU/L and 71.5 kUA/L, respectively) were elevated. The results for *Aspergillus fumigatus*-specific IgG were negative and the eosinophil count was high (1009/mm^3^). *Aspergillus fumigatus* was found in the mucus plug*.* She was treated with systemic corticosteroids.

## Discussion and conclusions

ABPA may present as an acute respiratory distress secondary to lung collapse [9–14]. Mucoid impaction may obstruct the lobar bronchi, leading to the finger-in-glove sign with lobar atelectasis seen on chest radiography. Lobar or segmental lung collapses have been described in 14–39% of patients with ABPA [6–8]. Our study showed that total lung collapse could also be a manifestation of ABPA although it is less frequently reported in the literature [9–16].

Interestingly, our cases were exclusively elderly women. However, no correlation has been reported between ABPA and the sex or age [19]. Moreover, 6 out of the 11 cases of total unilateral atelectasis described in the literature are male patients aged 6–74 years old [9, 10, 13, 15].

Not all of our five cases of total lung collapse due to ABPA met the criteria for ABPA, according to the latest definition of the ISHAM Working group [17] (Table 2). Indeed, total and specific IgE levels greater than 1000 IU/mL and 0.35 kUA/L, respectively, are both considered essential criteria [20, 21]. Agarwal et al. have found that only 39% of patients with ABPA met at least 7 out of the 8 components of the Rosenberg-Patterson criteria, including total serum IgE elevated, more than 1000 IU/mL [22]. In two out of our five cases, the total IgE levels were less than this threshold, as well as in two cases reported in the literature (Table 1). However, the relevance of the 1000 IU/mL threshold has been discussed by Saxena et al. A total IgE level greater than 500 IU/mL has been identified as an essential criteria associated with an improved diagnostic performance [23]. Moreover, if we used Asano et al. criteria for ABPA/ABPM [18] that have shown better sensitivity and specificity for the diagnosis of ABPA compared to the Rosenberg and Patterson criteria and to the ISHAM criteria, especially in atypical cases without asthma, all our patients could be considered as definite or probable cases of ABPA (Table 2).

**Table 1:** Cases of unilateral lung collapse secondary to ABPA reported in the literature [9–14, 26, 27]

Reference (first author, *journal*, year)	Patient’s age and underlying condition	Diagnosis of ABPA	Mechanic mold removal	Treatment
Brueton, *Arch Dis Child*, 1980	♂ 2 years old Cystic fibrosis	Skin prick test + Specific and total IgE Eosinophil count *Af* precipitins -	FB: no mucus plug	Oral corticosteroids
	♂ 6 years old Cystic fibrosis	Sputum: culture + skin prick test + Total IgE (500 IU/L) Eosinophil count Specific IgG +	FB: copious brown sputum aspirated from the right main bronchus	Physiotherapy and nebulized disodium cromoglycate and salbutamol
Berkin, *BMJ*, 1982	♀ 60 years old Cured pulmonary tuberculosis	BW: culture + skin prick test + Eosinophil count Specific IgG +	FB	Physiotherapy
	♂ 74 years old No history of respiratory disease	BW: culture + skin prick test + Eosinophil count Specific IgG +	Unsuccessful	Physiotherapy Flucytosine
Bhagat, *J Allergy Clin Immunol,* 1993	♀ 38 years old Left lower lobectomy for severe hemoptysis (aspergilloma in a bronchiectatic cavity)	Total IgE (250 IU/L) Eosinophil count Positive type I and III tuberculin skin test *Af* precipitins +	Unsuccessful twice	Prednisolone
Nomura, *Nihon Kokyuki Gakkai Zasshi*, 1998	♂ 29 years old Asthma	BW: culture + skin prick test + Specific and total IgE Eosinophil count Specific IgG +	Unsuccessful	Fluconazole Systemic and inhaled steroids
Agarwal, *Mycoses*, 2009	♀ 22 years old No history of respiratory disease	BW: culture + Specific and total IgE Eosinophil count Specific IgG +	RB	Steroids
Braude, *Intern Med J*, 2011	♀ 85 years old No history of respiratory disease	BW: culture + Specific and total IgE Eosinophil count Specific IgG +	FB	Intravenous hydrocortisone
Ghosh, *BMJ*, 2012	♀ 68 years old No history of respiratory disease	*Af* in the bronchial biopsy Eosinophil count	Inefficient	Steroids Itraconazole
Kumar, *Pneumonol Alergol Pol*, 2015	♂ 45 years old Asthma	BW: culture + Positive skin prick test Specific and total IgE Eosinophil count Specific IgG +	MD	Steroids LABA
	♂ 62 years old No history of respiratory disease		MD	Steroids Itraconazole

BW: bronchial wash, LABA: long-acting beta2 agonists, RB: rigid bronchoscopy, FB: flexible bronchoscopy, MD: missing data

**Table 2:** Diagnostic criteria for ABPA used for the five cases

	Diagnostic criteria for ABPA proposed by the ISHAM working group					Diagnostic criteria for ABPA/ABPM proposed by Asano et al.^18^
	Obligatory criteria		Minor criteria			
	Elevated *Aspergillus*-specific IgE level (> 0.35 kUA/L)	Elevated serum total IgE level (> 1000 IU/mL or simply elevated if all other criteria are met)	Precipitating antibodies or IgG antibodies against *Aspergillus*	Radiographic findings consistent with ABPA*	Peripheral blood eosinophilia (≥ 500 cells/mm^3^)	Definite ABPA: ≥ 6 criteria met Probable ABPA: ≥ 5 criteria met
Case 1 70 years old	Yes (8.05 kU/L)	No (791 kU/L)	Positive for *Aspergillus fumigatus* precipitins (4 precipitin bands)	Total left lung collapse on X-ray	No (210/mm^3^)	Previous asthma-like symptoms Elevated total serum IgE level Specific IgE for filamentous fungi ^†^ Presence of specific IgG for filamentous fungi ^†^ Filamentous fungal growth in cultures ^†^ Presence of mucus plugs in central bronchi, based on bronchoscopy
Case 2 74 years old	Yes (62.6 kU/L)	Yes (3364 kU/L)	Positive for *Aspergillus*-specific IgG (94 kUA/L)	Total left lung collapse, central bronchiectasis and presence of mucus plugs in the central bronchi on CT	Yes (2500/mm^3^)	Peripheral blood eosinophilia (> 500 cells/mm^3^) Elevated total serum IgE level Immediate cutaneous hypersensitivity and specific IgE for filamentous fungi ^†^ Presence of specific IgG for filamentous fungi ^†^ Filamentous fungal growth in cultures ^†^ Presence of mucus plugs in central bronchi, based on CT/bronchoscopy
Case 3 73 years old	Yes (10.3 kU/L)	No (406 kU/L)	Negative	Total left lung collapse, no high-attenuation mucus plug or central bronchiectasis in the contralateral lung	Yes (800/mm^3^)	Current or previous asthma-like symptoms Peripheral blood eosinophilia (> 500 cells/mm^3^) Specific IgE for filamentous fungi ^†^ Presence of fungal hyphae in bronchial mucus plugs Presence of mucus plugs in central bronchi, based on CT/bronchoscopy and mucus plug expectoration history
Case 4 72 years old	Yes (56.6 kU/L)	Yes (2442 kU/L)	Positive	Complete atelectasis of the left lung, tram-track opacities in the right lower field	No**	Elevated total serum IgE level Specific IgE for filamentous fungi ^†^ Presence of specific IgG for filamentous fungi ^†^ Filamentous fungal growth in endoscopic cultures ^†^ Presence of mucus plugs in central bronchi, based on bronchoscopy
Case 5 81 years old	Yes (71.5 kUA/L)	Yes (2464 kU/L)	Negative	Right lung atelectasis on CT, no other findings consistent with ABPA	Yes (1009/mm^3^)	Current or previous asthma-like symptoms Peripheral blood eosinophilia (> 500 cells/mm^3^) Elevated total serum IgE level Specific IgE for filamentous fungi ^†^ Filamentous fungal growth in cultures ^†^ Presence of mucus plugs in central bronchi, based on bronchoscopy

ABPA: Allergic bronchopulmonary aspergillosis. ABPM: Allergic bronchopulmonary mycosis

*Radiographic findings consistent with ABPA may be transient (i.e., consolidation, nodules, tram-track opacities, toothpaste/finger-in-glove opacities, fleeting opacities) or permanent (i.e., parallel line and ring shadows, bronchiectasis and pleuropulmonary fibrosis)

**The only eosinophil count available was obtained while the patient was already treated with corticosteroids

^†^Filamentous fungus: *Aspergillus fumigatus*

Although cystic fibrosis and asthma are the main respiratory conditions usually associated with ABPA [3, 4], none of our patients had a significant history of respiratory disease or atopy. Our observation is consistent with the few cases of total unilateral atelectasis described in the literature and summarized in Table 1: only 6 out of the 11 patients had a significant respiratory history. Interestingly, in a cohort of 530 patients*,* Muthu et al*.* have suggested two phenotypes of ABPA defined according to the status of asthma (yes/no). Patients with ABPA and asthma were more symptomatic, experienced more exacerbations and had a worse lung function [20]. As reported in the study by Glancy et al*.*, in which 11 out of the 42 patients with allergic broncho-pulmonary fungal diseases did not have clinical asthma, the absence of an underlying respiratory condition made it difficult to establish the diagnosis of ABPA [24]. Patients sometimes underwent surgery before the diagnosis of ABPA could be made, in particular when a tumor was initially suspected.

In this context, the diagnosis of ABPA may be supported by the presence of typical radiologic findings. The most common CT-scan findings are mucoid impactions and bronchiectasis predominantly involving the segmental and subsegmental bronchi of the upper lobes, with centrilobular nodules [6–8]. In case of total unilateral lung collapse, these imaging findings should be investigated in the contralateral lung. A CT scan was performed in three of our patients. Unfortunately, only one had imaging findings consistent with ABPA in the contralateral lung that could have guided the diagnosis. In the other cases, bronchoscopy was first performed rather than a CT scan because the clinical and biological findings strongly suggested the diagnosis of ABPA, especially in the two patients with a history of ABPA.

The diagnosis of ABPA is always challenging in patients with total lung collapse, in the absence of a preexisting respiratory condition and other characteristic findings on the CT scan. Many criteria have been proposed over the years with different diagnostic algorithms [17, 25, 26] but there is a lack of consensus for standardized criteria. When a patient present with total lung collapse associated with clinical and biological findings consistent with ABPA, we recommend to use the 10-component diagnostic criteria for ABPA/ABPM proposed by Asano et al. [18]. Flexible bronchoscopy should be considered when lung collapse is associated with respiratory distress, with the objective of removing obstructive mucus plugs. Besides, the endoscopic aspect of the mucus may be helpful for the diagnosis, and Asano et al. have retained this criterion in their recent criteria for ABPA/ABPM. Interestingly, the use of a cryoprobe may increase the likelihood of successfully removing the plugs, as shown here in one patient.

In addition, the microscopic analysis of the plugs could be the cornerstone of the diagnosis of ABPA. Cytologic findings associate necrotic eosinophils, Charcot Leyden crystals, small numbers of degenerating fungal hyphae, and eosinophil-rich inflammation with isolation of *Aspergillus* spp. in the fungal culture [27]. Nevertheless, flexible bronchoscopy is usually associated with limited success, as shown in our cases. Even if therapeutic rigid bronchoscopy could be another option, the endoscopic removal of the mucus plugs was inefficient in 4 out of our 5 cases. In this situation, additional drug treatment is often needed, such as systemic corticosteroids that suppress the inflammatory activity and *Aspergillus* hypersensitivity [3, 21]. Corticosteroids alone could be sufficient but repeated bronchoscopy may be required once corticosteroids have been initiated. Prolonged therapy is often needed [24]. To limit glucocorticoid-related adverse effects, itraconazole may also be used [28]. Itraconazole was used in combination with steroids as a first-line treatment in 4 out of our 5 patients to achieve a steroid-sparing effect and to decrease the fungal load. As described by Agarwal et al., itraconazole alone may be considered for the management of acute ABPA, and we used it in one patient (case 3) [19]. Other azoles may be proposed as a second line but *Aspergillus* susceptibility to azoles needs to be assessed to choose the optimal treatment. Lastly, a home visit performed by a medical indoor environment counselor is helpful to identify the source of exposure and to provide advice to reduce *Aspergillus* exposure [29, 30].

In conclusion, ABPA can cause total unilateral lung collapse even in patients without underlying chronic respiratory disease, making the diagnosis challenging. Initial flexible bronchoscopy may fail to completely remove the mucus plugs but seems essential in case of respiratory distress. It is very helpful to make the diagnosis (typical aspect of the mucus plugs) and to rule out other conditions, especially a lung cancer. Medical treatment with corticosteroids should be initiated early to suppress immune hyperreactivity although well-designed trials using steroids in ABPA are lacking. However, rigid bronchoscopy should be considered in the second line and the use of a cryobiopsy probe may be helpful to rapidly remove large plugs.

## Data Availability

The datasets used and/or analysed during the current study are available from the corresponding author on reasonable request.

## Abbreviations

ABPA: Allergic bronchopulmonary aspergillosis
ABPM: Allergic bronchopulmonary mycosis
Af: *Aspergillus fumigatus*
BW: Bronchial wash
CT: Computed tomography
CRP: C-reactive protein
EC: Eosinophil count
FB: Flexible bronchoscopy
IgE: Immunoglobulin E
LABA: Long-acting beta2-agonists
MD: Missing data
RB: Rigid bronchoscopy
